# Use of Drugs Associated with QT Interval Prolongation at the Hospital Level during the COVID-19 Pandemic in Colombia

**DOI:** 10.1155/2022/3045942

**Published:** 2022-09-21

**Authors:** Andrés Gaviria-Mendoza, Manuel Enrique Machado-Duque, Luis Fernando Valladales-Restrepo, Carlos Fernando Tovar-Yepes, Jorge Enrique Machado-Alba

**Affiliations:** ^1^Grupo de Investigación en Farmacoepidemiología y Farmacovigilancia, Universidad Tecnologica de Pereira-Audifarma S.A, Calle 105 No. 14-140, Pereira, Risaralda, Colombia; ^2^Grupo de Investigación Biomedicina, Facultad de Medicina, Fundación Universitaria Autónoma de las Américas, Ave Las Americas #98-56, Pereira, Colombia

## Abstract

**Background:**

Many of the therapeutic proposals for COVID-19 have been associated with adverse effects, including the risk of QT interval prolongation and torsades de pointes (TdP). The objective was to determine the use of drugs with a risk of QT interval prolongation in 21 clinics/hospitals in Colombia from January to December 2020.

**Methods:**

This cross-sectional study identified drug use according to pharmacological groups with potential risk of QT interval prolongation according to a risk classification: conditional, possible, and known risk of TdP. Descriptive analyses were performed.

**Results:**

A total of 355,574 patients who received QT-prolonging drugs were identified (equivalent to 51.4% of all inpatients treated during the study period). Of the group of patients on QT drugs, 54.4% used at least one drug with conditional risk, 52.6% with possible risk, and 40.3% with known risk. The most commonly used belonged to the group of drugs for the nervous system (63.0%), alimentary tract and metabolism (56.8%), anti-infectives for systemic use (13.0%), and the cardiovascular system (11.7%). On average, patients received 2.0 ± 1.5 risk drugs. Regarding drugs initially considered against COVID-19, 2,120 patients (0.6%) received azithromycin, 802 (0.2%) received chloroquine, 517 received hydroxychloroquine (0.1%), and 265 received lopinavir/ritonavir (0.1%).

**Conclusion:**

The high proportion of patients treated at the hospital level who receive drugs with risk of prolonging the QT interval should alert those responsible for their care to avoid fatal outcomes, especially during the COVID-19 epidemic, when some QT drugs are being used more frequently.

## 1. Introduction

Since the emergence and subsequent spread of severe acute respiratory syndrome coronavirus 2 (SARS-CoV-2), which causes coronavirus disease 2019 (COVID-19), multiple treatment proposals have been generated based mainly on data from in vitro studies and experiences with previous virus epidemics with similar characteristics, such as the Middle East respiratory syndrome (MERS) coronavirus and the SARS coronavirus [1, 2]. However, the current therapeutic approach for COVID-19 is basically summarized as support measures and symptom management, while individuals with more aggressive clinical presentations may require ventilatory support [1, 3].

Several hypotheses about the pathophysiology of COVID-19 have allowed us to propose various therapies. Because of the cytokine storm associated with COVID-19, molecules capable of regulating the body's immune response to SARS-CoV-2 infection, such as interleukin 6 or interleukin 1 inhibitors, have been mentioned [4, 5]. Antivirals have also been obvious options, for example, lopinavir/ritonavir presented early promising results when used together with other molecules [6]. These results were not confirmed by subsequent trials, and these molecules are not currently recommended in clinical practice guidelines [3]. However, the drugs that attracted the most attention at the beginning of the pandemic were hydroxychloroquine and chloroquine [1, 7], and these were initially included in some management guidelines [8].

In vitro analyses have indicated that hydroxychloroquine and chloroquine can prevent viral endocytosis [1]. Initially, in France, an open-label, nonrandomized clinical trial was conducted in which the authors concluded that hydroxychloroquine was associated with the reduction and disappearance of the viral load, with an effect reinforced by azithromycin [9], leading to great expectations and public suggestions that these two drugs would be a fundamental part of the treatment of COVID-19 [10].

Many of these therapeutic proposals have been associated with adverse effects, including at the cardiac level, such as QT interval prolongation, torsades de pointes (TdP) [11, 12], and myocardial injury or blocks, many of which are potentially fatal [13]. In the hospital setting, it has been described that 21% of patients experience QT interval prolongation one week after admission [14]. In Colombia, it has been estimated that up to 11% of outpatients older than 65 years receive some drug with a risk of prolonging the QT interval [15]. However, reports on the use of drugs with a risk of QT prolongation at the inhospital level in the regional context are scarce. This study sought to determine the use of drugs with a risk of QT prolongation in 21 hospital institutions in Colombia from January to December 2020.

## 2. Material and Methods

### 2.1. Design, Patients, and Data Collection

A descriptive observational study was conducted in which data on drug dispensing from January 1 to December 31, 2020 in 21 tertiary and quaternary (high complexity) hospitals and clinics in Colombia were reviewed. The data were obtained from the database of the company responsible for drug dispensing (Audifarma SA) in the hospital pharmacies for each of the institutions.

The records of all patients treated during the study time were included, and the following variables were analyzed: time of dispensing (month), sex, clinic or hospital, city, and drugs with a potential risk of causing QT interval prolongation.

The risk of drugs with a potential of prolonging the QT interval was categorized into conditional (0.25 points), possible (0.5 points), and known (3 points) risk according to the classification by http://Crediblemeds.org [16–18] and to the scores assigned in the corrected QT (QTc) interval prolongation scale (RISQ-PATH) [19, 20]. Drugs with conditional risk were those that have been associated with TdP but only when used concomitantly with other drugs at risk of prolonging the QT interval or in patients with risk conditions or comorbidities. The possible risk category includes drugs that can cause QT prolongation but have not yet been shown to cause TdP at the usual doses. Finally, known risk includes drugs that prolong the QT interval, and their relationship with TdP is clearly described, even when used according to the recommendations [16, 18].

Topical presentations of risk drugs (such as ophthalmic solutions or ointments) were excluded from analysis, except for buprenorphine patch. In addition, the specific consumption of chloroquine, hydroxychloroquine, and azithromycin, which are drugs with known risk (3 points), and of lopinavir/ritonavir, whose risk is possible (0.5 points), were verified. The list of identified drugs and their respective risk score and Anatomical Therapeutic Chemical (ATC) classification can be found in Annex 1.

For each patient, the sum of the scores was calculated based solely on the risk values for each drug. The other covariates of the RISQ-PATH score, such as age, sex, body mass index, presence of comorbidities, or paraclinical values, were not analyzed.

### 2.2. Baseline Characteristics

In order to compare the data obtained during the study period, the general drug consumption figures (aggregates, in general, and by risk classification) were collected for the study hospitals during the year 2019.

### 2.3. Statistical Analysis

The statistical package SPSS version 26.0 for Windows (IBM, USA) was used for data analysis. The use of drugs that can prolong the QT interval according to their level of risk, sum of scores, per month of use, and their proportion of use according to covariates and pharmacological groups were described.

## 3. Results

### 3.1. Total Number of Treated Patients

The 21 hospital centers evaluated were distributed among 11 different cities in Colombia, mainly Bogotá (*n* = 5; 23.8%), Medellín (*n* = 4; 19.0%), and Pereira (*n* = 3; 14.3%). Fourteen institutions were tertiary (66.7%), and seven were quaternary (33.3%) level care centers. Considering all hospital centers together, an average of 67,917 people were treated each month, decreasing from 90,872 in January to 40,191 in April (-55.8% variation compared to the January), and ending in December with 69,526 people (Table 1). The monthly average per institution was 3,234 treated patients (range: 1,913-4,381 patients) during the year of study.

### 3.2. Drugs with A Risk of QT Interval Prolongation

During the study period, a total of 2,159,785 dispensing records were reviewed, equivalent to 355,574 patients receiving drugs with a potential risk of QT interval prolongation (the same patient could be treated in more than one different month of observation). Of these, 54.3% were women (*n* = 192,983).

For the month of December, the percentage of patients who received at least one drug with a risk of QT interval prolongation varied among the different institutions, from 28.3% to 69.9% (Table 1). During the study period and among the total institutions evaluated, 51.4% of patients received drugs that could prolong the QT interval (53.2% considering only the data from March to December 2020). The 50.3% of patients treated in tertiary institutions received drugs with a risk of QT interval prolongation, compared to 53.7% in the quaternary level of care.

The total number of patients treated decreased since March, both overall at each institution and specifically with respect to patients receiving drugs with a risk of QT interval prolongation. However, the proportion of patients using these drugs remained stable during the first three months and then increased since April (Table 1).

Additionally, for the year 2019, the general mean in the percentage of patients that received drugs that could prolong the QT interval was 47.5%. This baseline data is shown in Annex 2 for each of the study hospitals.

### 3.3. Risk Classification

**Figure 1:**
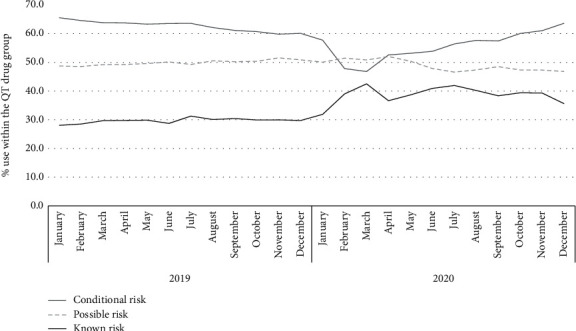
Proportion of patients with QT-prolonging drugs according to risk category, Colombia, 2019–2020.

During the study year, 54.4% (*n* = 193,528) of the patients received QT drugs with conditional risk, 52.6% (*n* = 187,042) with possible risk, and 40.3% (*n* = 143,292) with known risk. The distribution according to risk levels for each month of study and during baseline is shown in Figure 1. It is noted that the proportion of patients receiving known risk drugs, among patients who received any risk drug, remained stable during baseline, and then increased in 2020 from 31.9% in January to 42.5% in March and ended at 35.6% in December. Of the 193,528 patients who used conditional risk drugs, a total of 110,147 also used some possible or known risk drug (56.9% of those receiving conditional risk drugs).

Table 2 shows the distribution of use of each pharmacological group, with the most commonly used being nervous system drugs (*n* = 224,140; 63.0%), alimentary tract and metabolism drugs (*n* = 202,046; 56.8%), anti-infectives in general for systemic use (*n* = 46,238; 13.0%), and cardiovascular system drugs (*n* = 41,564; 11.7%). Notably, the subgroup of psychotropic drugs (antiparkinsonians, psycholeptics, and psychoanaleptics) accounted for 11.1% (*n* = 39,582), and antibiotics (antibacterials for systemic use) accounted for 10.0% (*n* = 35,683).

Regarding each molecule in particular, the most commonly used drugs were tramadol, omeprazole, and ondansetron. A detailed list of use of each drug is provided in Table 3. The main drugs used within the known risk group included ondansetron, propofol, haloperidol, clarithromycin, ciprofloxacin, and amiodarone (Table 3).

On average, patients received 2.0 ± 1.5 risk drugs (median: 1.0; interquartile range [IQR]: 1.0-2.0). A total of 52.4% (*n* = 186,399) used only one drug, while 24.0% used two (*n* = 85,187), 12.1% used three (*n* = 42,924), 5.6% used four (*n* = 19,804), and 5.9% (*n* = 21,260) used five or more risk drugs.

The mean sum of the QT risk score including only the values of the drugs (without considering other risks or comorbidities) was 2.0 ± 2.2 points, with a median of 0.75 (IQR: 0.5–3.3) points. The mode of the total score was 0.5 (*n* = 101,498; 28.5%). The 1.0% (*n* = 3,717) of patients had scores of 10 or more.

### 3.4. Therapies That Have Been Used in COVID-19

**Figure 2:**
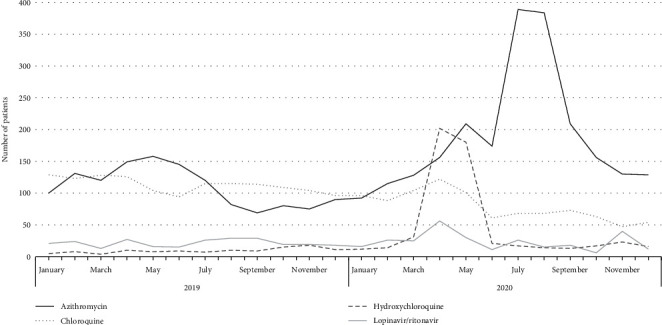
Patients treated with drugs initially proposed for the management of COVID-19 and that are associated with risk of QT interval prolongation in 21 hospital institutions, Colombia, 2019–2020.

During the study period, a total of 2,120 patients received azithromycin (0.6%), 802 chloroquine (0.2%), 517 hydroxychloroquine (0.1%), and 265 lopinavir/ritonavir (0.1%). Although the use of these drugs was proportionally low, the number of patients taking them increased after March. The number of patients exposed to azithromycin increased until August, while the other three molecules were used mainly in the period from March to June.  Figure 2 shows the variation in the number of patients receiving these drugs during the study period and during baseline.

## 4. Discussion

It was determined that more than 50% of patients treated at the hospital level received at least one drug with potential risk of prolonging the QT interval. This percentage was slightly higher during 2020 compared with the baseline from 2019, especially after March 2020. This finding should serve to alert prescribers and other caregivers, especially during the COVID-19 epidemic, when some risk drugs might be used more frequently.

The total number of patients treated at the institutions decreased during the study period, which may be explained by the context of the pandemic. However, the proportion of patients using drugs with a risk of prolonging the QT interval remained stable, emphasizing the importance of evaluating this type of risk in each hospitalized patient, not only during the pandemic.

Reports on the proportion of use of drugs with a risk of QT interval prolongation at the hospital level are scarce. For example, a study in Germany conducted a similar analysis but in elderly patients discharged from geriatric units [21]; in this population, 59% received at least one drug with a risk of QT interval prolongation, a rate higher than that identified in the present study [21]. Another study in Italy also found a higher proportion of use of drugs with a risk of QT interval prolongation (>89%) [22]. However, in those studies, the included population consisted only of older adults, not of the general population treated.

Other studies that address hospitalized patients focus on specific cases with long QT syndrome. A study in a geriatric hospital unit in France showed that 22% of the treated patients had prolonged QT interval values, with men and those using drugs classified as with risk for QT interval prolongation presenting higher risk [23]. In Paraguay, it was determined that up to 21% of patients older than 16 years treated in two institutions in 2019 displayed prolonged QT syndrome during hospitalization; the most frequently used risk drugs were omeprazole, furosemide, piperacillin-tazobactam, tramadol, and ondansetron [14], similar to the present analysis.

The high proportion of risk drugs in the nervous system group is explained by the fact that this group includes drugs typically used at the hospital level, such as opioids and general anesthetics. Likewise, a high proportion of drugs from the alimentary tract group was found (mainly due to the use of proton pump inhibitors). Important interventions could be carried out to promote the rational prescription and reduced use of this group of drugs, given that previous studies have found an unjustified use close to 50% in hospitalized patients [24].

Given that approximately 11% of patients older than 65 in Colombia receive drugs with risk of QT interval prolongation at the outpatient level [15], and that this age group is particularly sensitive to adverse or fatal outcomes in case of SARS-CoV-2 infection [8], exposure to the therapies with a risk of prolonging the QT interval proposed for the management of COVID-19 makes these patients more susceptible. Mercuro et al. described a cohort of 90 patients with COVID-19 who received hydroxychloroquine (approximately 60% in combination with azithromycin) and found that 20% had QTc values ≥500 milliseconds after starting treatment, and a case of TdP was reported; in addition, 73.3% used two or more drugs with risk of QT interval prolongation [12]. In this study, more than 45% of patients used two or more risk drugs, indicating that in the treatment of patients with COVID-19, it would be very likely that the number of patients receiving risk drugs would be maintained or increase.

The possible relationship between the increased probability of occurrence of QT interval prolongation and using a greater number of risk drugs has been previously described [25]. In fact, this is part of the RISQ-PATH score [19, 20]. In the present study, it was only possible to assign a score according to the classification of each drug as conditional, possible or known risk, finding a mean of 2 points. Although this classification indicates that patients with less than 10 points have a low risk of QT interval prolongation [20], other unmeasured variables that may increase the individual risk of patients should be considered, including age 65 years or older, smoking, and presence of hypertension or ischemic heart disease (3 points each) [19, 20]; these unmeasured variables represent a limitation of the present study. Many of these risks also coincide with those described for the development of adverse outcomes in patients with COVID-19, as previously mentioned.

In the study by Mercuro et al., it was also found that the use of loop diuretics was associated with an increased risk of QTc ≥ 500 milliseconds [12]. In the present analysis, more than 10% of the QT drugs were diuretics, highlighting the importance of considering the cardiovascular risk of these drugs at the hospital level. Regarding the use of drugs of known risk, a proportion close to 40% was found, which is higher than that described in a group of patients treated at a coronary unit (28%); this may be explained by the fact that the cited study did not report the use of propofol, which was the second most frequently used risk drug in the present study [25].

Although the use of azithromycin was low, it should be considered that other antibiotics frequently used in hospitalized patients may also present a risk of QT interval prolongation, for example, piperacillin-tazobactam. Patients with severe COVID-19 may have clinical characteristics that make it difficult for physicians to determine the presence of a concomitant bacterial infection; therefore, an increase in the use of antibiotics during the pandemic has been described, although it was not necessary in many patients [26, 27]. This can increase not only the risk of QT interval prolongation but also of other adverse events and resistance to antibiotics [26].

Hydroxychloroquine, chloroquine, and lopinavir/ritonavir had relatively low consumption with respect to the total QT risk drugs. However, their use was higher during the beginning of the pandemic. It is important to monitor likely increases in adverse reactions. In this sense, a pharmacovigilance network in France described an increase in adverse cardiac reactions (especially QTc prolongation) associated with these drugs during the pandemic [11]. So far, none of these therapies have been able to demonstrate a real benefit for the management of the disease [28–30], and their use in Colombia is not supported by the local infectiology association or government agencies [3, 31, 32].

For other drugs proposed for COVID-19, such as remdesivir, there is still not sufficient information to define whether they are associated with an increased risk of QT interval prolongation; therefore, their cautious use is essential [13, 16, 33]. Other drugs, such as favipiravir, already have some identified relationship with prolonged QT interval [33]; and on the contrary, tocilizumab has been associated with decreased QT interval [13, 18].

Caution should be exercised in the management of COVID-19 when formulating drugs traditionally used for other diseases because these would have a demonstrated safety and efficacy profile in conditions that cannot be entirely extrapolated to the current circumstances of patients infected by SARS-CoV-2. During the current pandemic, it has been possible to propose and quickly start clinical trials that test these therapies, and both physicians and researchers should be aware of the frequent changes in recommendations that may be made regarding effectiveness and safety profiles [34]. These changes and updates in clinical practice guidelines may explain the transient use of azithromycin and the other drugs initially proposed for COVID-19 seen in the current study.

Considering that several of the therapies proposed for COVID-19 are associated with cardiovascular risks and arrhythmias with prolongation of the QT interval, management algorithms have been generated to mitigate their impact, which include performing a baseline electrocardiogram, measuring the QTc, determining calcium, magnesium and potassium levels, and verifying the use of QT risk drugs before starting their use [35, 36]. Similar recommendations were already described when routinely using risk drugs in the hospital environment before the pandemic [37]. These aspects were not evaluated in the present study, and it would be important to conduct new studies that analyze prescriptions in detail, including safety measures and monitoring.

This study has other limitations secondary to its observational design. Some variables of interest were not available, such as the age of the treated patients, comorbidities, and the detailed classification of each care service and their respective number of beds. In addition, the comedications received by patients that were not directly related to prolongation of the QT interval and that could affect the risk of complications were not verified. Additionally, diagnoses were not reviewed (for example, to verify the total cases of COVID-19 cases attended in the study institutions or the specific use of drugs intended for COVID-19 management) nor were reports of adverse reactions. Baseline characteristics were only available as aggregates, and it was not possible to analyze 2019 data in detail. Lastly, electrocardiogram reports were also not available. Further studies describing the use of these particular therapies are needed.

## 5. Conclusion

Approximately half of the patients treated in 21 hospital centers used at least one risk drug, and of these, more than 45% received various drugs that increase the probability of QT interval prolongation. Health workers should be aware of this level of risk, especially when considering the use of proposed new molecules for the treatment of COVID-19 that may also increase this cardiac risk.

## Figures and Tables

**Table 1:** Percentage of use of medications with risk of QT interval prolongation in 21 hospital institutions, Colombia, January–December 2020.

Institution	City	Health care complexity (level)	QT patients (%)^∗^												
			January	February	March	April	May	June	July	August	September	October	November	December	Variation^∗∗^
1	Cali	3	63.4	57.5	55.6	64.8	59.6	59.9	62.4	64.5	67.3	84.6	67.8	63.5	0.2%
2	Popayán	3	56.6	56.4	56.7	63.8	61.7	60.7	59.6	57.1	56.8	79.3	58.3	58.4	3.2%
3	Cartagena	3	56.0	52.8	61.9	73.5	73.8	66.8	63.6	69.0	67.3	81.8	56.8	69.9	24.8%
4	Buga	3	49.7	45.4	46.7	48.6	47.6	49.9	51.9	50.9	48.0	51.7	51.0	54.1	8.9%
5	Medellín	3	48.5	49.5	46.8	52.4	45.9	46.4	47.0	51.1	51.0	50.4	50.2	50.3	3.7%
6	Manizales	3	48.0	44.8	44.0	47.8	50.9	51.2	52.8	56.1	53.4	72.9	51.7	49.0	2.1%
7	Manizales	3	46.9	48.3	46.1	53.3	56.1	54.9	54.3	56.1	57.4	53.2	54.5	52.6	12.2%
8	Barranquilla	3	46.1	44.2	44.9	52.2	52.7	59.7	61.7	63.4	62.4	62.1	65.8	60.5	31.2%
9	Armenia	3	42.5	42.7	44.2	50.6	45.5	45.3	45.8	50.1	48.6	50.0	49.6	50.3	18.4%
10	Ibagué	3	41.8	40.5	39.7	45.3	47.5	43.9	46.0	47.7	47.6	49.5	50.4	53.7	28.5%
11	Bogotá	3	37.7	32.6	35.0	46.8	44.7	46.5	43.7	48.9	48.1	50.4	52.6	49.1	30.2%
12	Pereira	3	37.6	37.2	38.6	69.9	NA	39.1	39.4	39.8	40.6	57.3	44.3	39.6	5.3%
13	Medellín	3	36.3	37.4	38.2	44.8	43.0	35.3	34.9	47.6	39.1	41.3	41.7	38.5	6.1%
14	Bogotá	3	25.3	20.3	29.1	38.7	36.8	34.7	33.3	33.6	32.6	32.5	27.5	28.3	11.9%
15	Medellín	4	56.8	55.9	58.7	63.4	61.2	71.6	68.9	63.4	60.9	60.3	62.4	62.0	9.2%
16	Medellín	4	54.5	52.6	52.3	60.0	60.9	58.9	58.0	58.8	52.6	53.6	57.4	57.2	5.0%
17	Bogotá	4	49.4	45.5	49.6	55.1	58.2	56.9	69.2	71.7	57.2	55.4	54.3	51.6	4.5%
18	Pereira	4	48.5	46.9	45.4	50.0	49.0	52.0	52.9	51.0	50.9	57.0	53.8	52.4	8.0%
19	Bogotá	4	48.0	44.6	46.3	51.5	54.2	53.1	51.2	55.4	53.6	54.6	55.0	51.5	7.3%
20	Pereira	4	47.8	46.7	48.4	53.9	54.5	54.9	58.1	55.7	55.1	60.3	58.1	55.5	16.1%
21	Bogotá	4	44.2	41.8	42.4	38.8	44.1	46.8	45.6	46.6	48.1	47.8	48.1	46.0	4.1%
Mean			46.9	44.9	46.2	53.6	52.4	51.8	52.4	54.2	52.3	57.4	52.9	52.1	11.0%
Total QT patients			42796	41179	33692	20828	27606	30802	32882	33848	37825	40053	37521	36264	-15.3%
Total patients attended			90872	92013	73320	40191	53176	58500	61774	62127	71903	71455	70141	69526	-23.5%

^∗^: Percentage of patients with at least one QT-prolonging drug. ^∗∗^: December vs January.

**Table 2:** Distribution of use of each pharmacological group of QT-prolonging drugs.

Medication group	Total patients (*n* = 355,574)	% within the QT group
Nervous system	224140	63.0
Opioids	164651	46.3
Anesthetics, general	62951	17.7
Psychotropic drugs (antiparkinson drugs, psycholeptics, and psychoanaleptics)	39582	11.1
Alimentary tract and metabolism	202046	56.8
Anti-infectives for systemic use	46238	13.0
Antibacterials for systemic use	35683	10.0
Antimycotics for systemic use	3611	1.0
HIV (antivirals for systemic use)	556	0.2
Cardiovascular system	41564	11.7
Diuretics	40098	11.3
Antiarrhythmics	3794	1.1
Systemic hormonal preparations, excluding sex hormones	30850	8.7
Respiratory system	9376	2.6
Antiparasitic products, insecticides, and repellents	4051	1.1
Antimalarials	1308	0.4
Antineoplastic and immunomodulating agents	1513	0.4
Blood and blood-forming organs	157	0.0
Musculoskeletal system	57	0.0
Genito-urinary system and sex hormones	61	0.0
Total	355574	100.0

**Table 3:** QT-prolonging drugs used in 355,574 patients in 21 hospital institutions, Colombia, January–December 2020.

Medications	Total patients (*n* = 355,574)	% within the QT group
Known risk	143292	40.3
Ondansetron	74017	20.2
Propofol	60475	17.1
Haloperidol	12659	3.4
Clarithromycin	11660	2.8
Ciprofloxacin	7102	2.1
Amiodarone	3784	1.0
Fluconazole	3375	0.9
Azithromycin	2120	0.6
Escitalopram	1934	0.5
Levomepromazine	1635	0.4
Methadone	850	0.2
Chloroquine	802	0.3
Erythromycin	740	0.2
Hydroxychloroquine	517	0.2
Sevoflurane	357	0.1
Domperidone	191	0.1
Terlipressin	187	0.1
Moxifloxacin	169	0.1
Oxaliplatin	166	0.0
Cilostazol	157	0.1
Levofloxacin	128	0.0
Donepezil	28	0.0
Arsenic trioxide	6	0.0
Possible risk	187042	52.6
Tramadol	163686	46.6
Oxytocin	30656	8.5
Dexmedetomidine	4552	0.8
Imipramine	962	0.3
Buprenorphine	934	0.3
Clozapine	735	0.2
Leuprolide	383	0.1
Lopinavir/ritonavir	265	0.1
Efavirenz	221	0.1
Lithium carbonate	210	0.1
5-fluorouracil	155	0.0
Tacrolimus	152	0.0
Bortezomib	147	0.0
Fingolimod	137	0.0
Norfloxacin	136	0.0
Capecitabine	132	0.0
Dasatinib	82	0.0
Tizanidine	57	0.0
Tolterodine	55	0.0
Nilotinib	53	0.0
Memantine	53	0.0
Degarelix	50	0.0
Aripiprazole	41	0.0
Venlafaxine	37	0.0
Mirtazapine	32	0.0
Tamoxifen	30	0.0
Primaquine	29	0.0
Bendamustine	29	0.0
Sorafenib	23	0.0
Pazopanib	22	0.0
Sunitinib	14	0.0
Crizotinib	12	0.0
Epirubicin	11	0.0
Dabrafenib	11	0.0
Osimertinib	10	0.0
Pasireotide	7	0.0
Mirabegron	7	0.0
Ribociclib	5	0.0
Bosutinib	3	0.0
Palonosetron	2	0.0
Paliperidone	1	0.0
Conditional risk	193528	54.4
Omeprazole	109947	26.0
Metoclopramide	54274	14.8
Furosemide	31598	9.0
Esomeprazole	20550	5.3
Piperacillin/tazobactam	18673	5.2
Metronidazole	14908	4.3
Hydrochlorothiazide	10696	2.9
Trazodone	10486	2.9
Diphenhydramine	9376	2.9
Quetiapine	7638	1.7
Sertraline	5460	1.5
Hydroxyzine	5413	1.5
Loperamide	3286	1.0
Amitriptyline	2425	0.7
Fluoxetine	2079	0.6
Risperidone	1357	0.4
Olanzapine	725	0.2
Amphotericin b	301	0.1
Amantadine	253	0.1
Pantoprazole	170	0.0
Lansoprazole	135	0.0
Voriconazole	120	0.0
Indapamide	81	0.0
Paroxetine	75	0.0
Atazanavir	59	0.0
Abiraterone	42	0.0
Ivabradine	37	0.0
Itraconazole	35	0.0
Posaconazole	33	0.0
Ketoconazole	29	0.0
Fluvoxamine	26	0.0
Atazanavir/ritonavir	26	0.0
Propafenone	18	0.0

## Data Availability

Name of repository: protocols.io code availability to repository data set: https://doi.org/10.17504/protocols.io.bxagpibw

## Supplementary Materials

Supplementary 1 Annex 1: the list of identified drugs and their respective risk score and Anatomical Therapeutic Chemical (ATC) classification.

Supplementary 2 Annex 2: percentage of use of medications with risk of QT interval prolongation in 21 hospital institutions, Colombia, January–December 2019.

## References

[1] Sanders J. M., Monogue M. L., Jodlowski T. Z., Cutrell J. B. (2020). Pharmacologic treatments for coronavirus disease 2019 (COVID-19). *Journal of the American Medical Association*.

[2] Zhai P., Ding Y., Wu X., Long J., Zhong Y., Li Y. (2020). The epidemiology, diagnosis and treatment of COVID-19. *International Journal of Antimicrobial Agents*.

[3] Saavedra Trujillo C. H., Sección V. (2021). Manejo del paciente con infección por SARS-CoV-2/COVID-19. *Infection*.

[4] Li H., Liu L., Zhang D. (2020). SARS-CoV-2 and viral sepsis: observations and hypotheses. *Lancet*.

[5] Liu B., Li M., Zhou Z., Guan X., Xiang Y. (2020). Can we use interleukin-6 (IL-6) blockade for coronavirus disease 2019 (COVID-19)-induced cytokine release syndrome (CRS)?. *Journal of Autoimmunity*.

[6] Hung I. F.-N., Lung K.-C., Tso E. Y.-K. (2020). Triple combination of interferon beta-1b, lopinavir-ritonavir, and ribavirin in the treatment of patients admitted to hospital with COVID-19: an open- label, randomised, phase 2 trial. *Lancet*.

[7] Cunningham A. C., Goh H. P., Koh D. (2020). Treatment of COVID-19: old tricks for new challenges. *Critical Care*.

[8] Saavedra Trujillo C. H. (2020). Consenso colombiano de atención, diagnóstico y manejo de la infección por SARS-COV-2/COVID 19 en establecimientos de atención de la salud. Recomendaciones basadas en consenso de expertos e informadas en la evidencia. *Infection*.

[9] Gautret P., Lagier J. C., Parola P. (2020). Hydroxychloroquine and azithromycin as a treatment of COVID-19: results of an open-label non-randomized clinical trial. *International Journal of Antimicrobial Agents*.

[10] Liu M., Caputi T. L., Dredze M., Kesselheim A. S., Ayers J. W. (2020). Internet searches for unproven COVID-19 therapies in the United States. *JAMA Internal Medicine*.

[11] Gérard A., Romani S., Fresse A. (2020). "Off-label" use of hydroxychloroquine, azithromycin, lopinavir-ritonavir and chloroquine in COVID-19: a survey of cardiac adverse drug reactions by the French network of pharmacovigilance centers. *Therapie*.

[12] Mercuro N. J., Yen C. F., Shim D. J. (2020). Risk of QT interval prolongation associated with use of hydroxychloroquine with or without concomitant azithromycin among hospitalized patients testing positive for coronavirus disease 2019 (COVID-19). *JAMA Cardiology*.

[13] Naksuk N., Lazar S., Peeraphatdit T. B. (2020). Cardiac safety of off-label COVID-19 drug therapy: a review and proposed monitoring protocol. *European Heart Journal Acute Cardiovascular Care*.

[14] Roy T., Peralta Giménez R., Gamarra Cardozo L. C., Núñez Vera L. M., Santacruz Sosa M. C. (2020). Prolonged QTc interval in patients of clinical medicine: multicenter study. *Revista Virtual de la Sociedad Paraguaya de Medicina Interna*.

[15] Moreno‐Gutiérrez P. A., Gaviria‐Mendoza A., Cañón M. M., Machado‐Alba J. E. (2016). High prevalence of risk factors in elderly patients using drugs associated with acquired torsades de pointes chronically in Colombia. *British Journal of Clinical Pharmacology*.

[16] AZCERT http://CredibleMeds.org.

[17] Schwartz P. J., Woosley R. L. (2016). Predicting the unpredictable: drug-induced QT prolongation and torsades de pointes. *Journal of the American College of Cardiology*.

[18] Woosley R. L., Romero K. (2013). Assessing cardiovascular drug safety for clinical decision-making. *Nature Reviews. Cardiology*.

[19] Buss V. H., Lee K., Naunton M., Peterson G., Kosari S. (2018). Identification of patients at-risk of QT interval prolongation during medication reviews: a missed opportunity?. *Journal of Clinical Medicine*.

[20] Vandael E., Vandenberk B., Vandenberghe J., Spriet I., Willems R., Foulon V. (2017). Development of a risk score for QTc-prolongation: the RISQ-PATH study. *International Journal of Clinical Pharmacy*.

[21] Schächtele S., Tümena T., Gaßmann K. G., Fromm M. F., Maas R. (2016). Co-prescription of QT-interval prolonging drugs: an analysis in a large cohort of geriatric patients. *PLoS One*.

[22] Rossi M., Marzi F., Natale M. (2021). Drug-associated QTc prolongation in geriatric hospitalized patients: a cross-sectional study in internal medicine. *Drugs Real World Outcomes*.

[23] Maison O., de la Gastine B., Dayot L., Goutelle S. (2017). Prevalence and risk factors of drug-associated corrected QT prolongation in elderly hospitalized patients: results of a retrospective analysis of data obtained over 6 months. *Drugs & Aging*.

[24] Machado-Alba J. E., Castrillón-Spitia J. D., Londoño-Builes M. J. (2014). An economic analysis of inadequate prescription of antiulcer medications for in-hospital patients at a third level institution in Colombia. *Revista Española de Enfermedades Digestivas*.

[25] Khan Q., Ismail M., Haider I. (2018). High prevalence of the risk factors for QT interval prolongation and associated drug-drug interactions in coronary care units. *Postgraduate Medicine*.

[26] Hsu J. (2020). How COVID-19 is accelerating the threat of antimicrobial resistance. *BMJ*.

[27] Rawson T. M., Moore L. S. P., Zhu N. (2020). Bacterial and fungal coinfection in individuals with coronavirus: a rapid review to support COVID-19 antimicrobial *Prescribing*. *Clinical Infectious Diseases*.

[28] Self W. H., Semler M. W., Leither L. M. (2020). Effect of hydroxychloroquine on clinical status at 14 days in hospitalized patients with COVID-19: a randomized clinical trial. *Journal of the American Medical Association*.

[29] WHO Solidarity Trial Consortium (2021). Repurposed antiviral drugs for Covid-19-interim WHO solidarity trial results. *The New England Journal of Medicine*.

[30] Cavalcanti A. B., Zampieri F. G., Rosa R. G. (2020). Hydroxychloroquine with or without azithromycin in mild-to-moderate COVID-19. *The New England Journal of Medicine*.

[31] Instituto Nacional de Vigilancia de Medicamentos y Alimentos-INVIMA (2020). *Listado de medicamentos priorizados para uso en pacientes que cursan con COVID-19*.

[32] Instituto Nacional de Vigilancia de Medicamentos y Alimentos-INVIMA (2020). *Acta no. 03 de 2020 (15 abril de 2020), sala especializada de moléculas nuevas, nuevas indicaciones y medicamentos biológicos*.

[33] Aggarwal G., Henry B. M., Aggarwal S., Bangalore S. (2020). Cardiovascular safety of potential drugs for the treatment of coronavirus disease 2019. *The American Journal of Cardiology*.

[34] Kalil A. C. (2020). Treating COVID-19-off-label drug use, compassionate use, and randomized clinical trials during pandemics. *Journal of the American Medical Association*.

[35] Giudicessi J. R., Noseworthy P. A., Friedman P. A., Ackerman M. J. (2020). Urgent guidance for navigating and circumventing the QTC-prolonging and torsadogenic potential of possible pharmacotherapies for coronavirus disease 19 (COVID-19). *Mayo Clinic Proceedings*.

[36] Sapp J. L., Alqarawi W., MacIntyre C. J. (2020). Guidance on minimizing risk of drug-induced ventricular arrhythmia during treatment of COVID-19: a statement from the canadian heart rhythm society. *The Canadian Journal of Cardiology*.

[37] Drew B. J., Ackerman M. J., Funk M. (2010). Prevention of torsade de pointes in hospital settings: a scientific statement from the American Heart Association and the American College of Cardiology Foundation. *Circulation*.

