# Supplementary material for: Use of Drugs Associated with QT Interval Prolongation at the Hospital Level during the COVID-19 Pandemic in Colombia
Source: Int J Vasc Med. 2022 Sep 21;2022:3045942. doi: 10.1155/2022/3045942 (PMC9520316; doi:10.1155/2022/3045942)
Supplement: Supplementary 1 — Annex 1: the list of identified drugs and their respective risk score and Anatomical Therapeutic Chemical (ATC) classification. [file 3045942.f1.docx]

**Annex 1**. QT-prolonging drugs used in the studied population, January-March 2020.

| **Drug** | **QT risk (score)** | **ATC Group** |
| --- | --- | --- |
| Abiraterone | 0.25 | Antineoplastic and immunomodulating agents |
| Amantadine | 0.25 | Nervous system |
| Amitriptiline | 0.25 | Nervous system |
| Amphotericin B | 0.25 | Antiinfectives for systemic use |
| Atazanavir | 0.25 | Antiinfectives for systemic use |
| Atazanavir / Ritonavir | 0.25 | Antiinfectives for systemic use |
| Diphenhydramine | 0.25 | Respiratory system |
| Esomeprazole | 0.25 | Alimentary tract and metabolism |
| Fluoxetine | 0.25 | Nervous system |
| Fluvoxamine | 0.25 | Nervous system |
| Furosemide | 0.25 | Cardiovascular system |
| Hydrochlorotiazide | 0.25 | Cardiovascular system |
| Hydroxycin | 0.25 | Nervous system |
| Indapamide | 0.25 | Cardiovascular system |
| Itraconazole | 0.25 | Antiinfectives for systemic use |
| Ivabradine | 0.25 | Cardiovascular system |
| Ketoconazole | 0.25 | Antiinfectives for systemic use |
| Lansoprazole | 0.25 | Alimentary tract and metabolism |
| Loperamide | 0.25 | Alimentary tract and metabolism |
| Metoclopramide | 0.25 | Alimentary tract and metabolism |
| Metronidazole | 0.25 | Antiinfectives for systemic use |
| Olanzapine | 0.25 | Nervous system |
| Omeprazole | 0.25 | Alimentary tract and metabolism |
| Pantoprazole | 0.25 | Alimentary tract and metabolism |
| Paroxetine | 0.25 | Nervous system |
| Piperacillin / Tazobactam | 0.25 | Antiinfectives for systemic use |
| Posaconazole | 0.25 | Antiinfectives for systemic use |
| Propafenone | 0.25 | Cardiovascular system |
| Quetiapine | 0.25 | Nervous system |
| Risperidone | 0.25 | Nervous system |
| Sertraline | 0.25 | Nervous system |
| Trazodone | 0.25 | Nervous system |
| Voriconazole | 0.25 | Antiinfectives for systemic use |
| 5-Fluorouracil | 0.5 | Antineoplastic and immunomodulating agents |
| Aripiprazole | 0.5 | Nervous system |
| Bendamustine | 0.5 | Antineoplastic and immunomodulating agents |
| Bortezomib | 0.5 | Antineoplastic and immunomodulating agents |
| Bosutinib | 0.5 | Antineoplastic and immunomodulating agents |
| Buprenorphine | 0.5 | Nervous system |
| Capecitabine | 0.5 | Antineoplastic and immunomodulating agents |
| Lithium Carbonate | 0.5 | Nervous system |
| Clozapine | 0.5 | Nervous system |
| Crizotinib | 0.5 | Antineoplastic and immunomodulating agents |
| Dabrafenib | 0.5 | Antineoplastic and immunomodulating agents |
| Dasatinib | 0.5 | Antineoplastic and immunomodulating agents |
| Degarelix | 0.5 | Antineoplastic and immunomodulating agents |
| Dexmedetomidine | 0.5 | Nervous system |
| Efavirenz | 0.5 | Antiinfectives for systemic use |
| Epirubicine | 0.5 | Antineoplastic and immunomodulating agents |
| Fingolimod | 0.5 | Antineoplastic and immunomodulating agents |
| Imipramine | 0.5 | Nervous system |
| Leuprolide | 0.5 | Antineoplastic and immunomodulating agents |
| Lopinavir / Ritonavir | 0.5 | Antiinfectives for systemic use |
| Memantine | 0.5 | Nervous system |
| Mirtazapine | 0.5 | Nervous system |
| Nilotinib | 0.5 | Antineoplastic and immunomodulating agents |
| Norfloxacin | 0.5 | Antiinfectives for systemic use |
| Osimertinib | 0.5 | Antineoplastic and immunomodulating agents |
| Oxytocin | 0.5 | Systemic hormonal preparations, excluding sex hormones |
| Palonosetron | 0.5 | Alimentary tract and metabolism |
| Pasireotide | 0.5 | Systemic hormonal preparations, excluding sex hormones |
| Pazopanib | 0.5 | Antineoplastic and immunomodulating agents |
| Primaquine | 0.5 | Antiparasitic products, insecticides and repellents |
| Ribociclib | 0.5 | Antineoplastic and immunomodulating agents |
| Sorafenib | 0.5 | Antineoplastic and immunomodulating agents |
| Sunitinib | 0.5 | Antineoplastic and immunomodulating agents |
| Tacrolimus | 0.5 | Antineoplastic and immunomodulating agents |
| Tamoxifen | 0.5 | Antineoplastic and immunomodulating agents |
| Tizanidine | 0.5 | Musculo-skeletal system |
| Tolterodine | 0.5 | Genito-urinary system and sex hormones |
| Tramadol | 0.5 | Nervous system |
| Venlafaxine | 0.5 | Nervous system |
| Amiodarone | 3 | Cardiovascular system |
| Azithromycin | 3 | Antiinfectives for systemic use |
| Cilostazole | 3 | Blood and blood forming organs |
| Cyprofloxacin | 3 | Antiinfectives for systemic use |
| Clarithromycin | 3 | Antiinfectives for systemic use |
| Chloroquine | 3 | Antiparasitic products, insecticides and repellents |
| Domperidone | 3 | Alimentary tract and metabolism |
| Donepezil | 3 | Nervous system |
| Erythromycin | 3 | Antiinfectives for systemic use |
| Escitalopram | 3 | Nervous system |
| Fluconazole | 3 | Antiinfectives for systemic use |
| Haloperidol | 3 | Nervous system |
| Hydroxychloroquine | 3 | Antiparasitic products, insecticides and repellents |
| Levofloxacin | 3 | Antiinfectives for systemic use |
| Levomepromazine | 3 | Nervous system |
| Methadone | 3 | Nervous system |
| Moxifloxacin | 3 | Antiinfectives for systemic use |
| Ondansetron | 3 | Alimentary tract and metabolism |
| Oxaliplatin | 3 | Antineoplastic and immunomodulating agents |
| Propofol | 3 | Nervous system |
| Sevoflurane | 3 | Nervous system |
| Terlipresin | 3 | Systemic hormonal preparations, excluding sex hormones |
| Arsenic Trioxide | 3 | Antineoplastic and immunomodulating agents |
