# Supplementary material for: Use of Drugs Associated with QT Interval Prolongation at the Hospital Level during the COVID-19 Pandemic in Colombia
Source: Int J Vasc Med. 2022 Sep 21;2022:3045942. doi: 10.1155/2022/3045942 (PMC9520316; doi:10.1155/2022/3045942)
Supplement: Supplementary 2 — Annex 2: percentage of use of medications with risk of QT interval prolongation in 21 hospital institutions, Colombia, January–December 2019. [file 3045942.f2.docx]

**Annex 2**. Percentage of use of medications with risk of QT interval prolongation in 21 hospital institutions, Colombia, January-December 2019.

| **Institution** | **City** | **Health care complexity (level)** | **QT patients (%)*** | | | | | | | | | | | | |
| --- | --- | --- | --- | --- | --- | --- | --- | --- | --- | --- | --- | --- | --- | --- | --- |
|  |  |  | **January** | **February** | **March** | **April** | **May** | **June** | **July** | **August** | **September** | **October** | **November** | **December** | **Variation**** |
| 1 | Cali | 3 | 60,6 | 57,5 | 58,6 | 59,9 | 60,4 | 63,5 | 62,5 | 60,4 | 60,4 | 62,2 | 64,4 | 66,2 | 9,2% |
| 2 | Popayán | 3 | 63,4 | 60,1 | 60,6 | 61,3 | 60,4 | 60,2 | 58,0 | 58,4 | 58,6 | 57,1 | 59,8 | 59,1 | -6,8% |
| 3 | Cartagena | 3 | 59,4 | 58,1 | 58,7 | 60,1 | 58,3 | 61,0 | 60,6 | 60,8 | 60,1 | 63,0 | 63,1 | 63,0 | 6,1% |
| 4 | Buga | 3 | 49,4 | 47,8 | 49,1 | 48,0 | 48,1 | 48,5 | 49,8 | 48,8 | 49,0 | 47,9 | 49,6 | 47,1 | -4,7% |
| 5 | Medellín | 3 | 46,2 | 47,6 | 48,4 | 50,8 | 48,5 | 49,8 | 47,1 | 46,5 | 48,9 | 48,5 | 49,1 | 46,7 | 1,1% |
| 6 | Manizales | 3 | 45,9 | 47,0 | 45,5 | 44,6 | 45,3 | 42,9 | 40,6 | 44,1 | 46,4 | 47,4 | 47,5 | 46,1 | 0,4% |
| 7 | Manizales | 3 | 47,4 | 46,0 | 46,6 | 45,1 | 45,8 | 47,8 | 47,4 | 48,1 | 48,2 | 47,4 | 47,2 | 49,1 | 3,6% |
| 8 | Barranquilla | 3 | 57,6 | 56,9 | 55,0 | 53,6 | 55,2 | 45,7 | 37,3 | 40,7 | 44,2 | 44,8 | 47,1 | 48,0 | -16,7% |
| 9 | Armenia | 3 | 44,1 | 45,3 | 42,4 | 37,3 | 40,4 | 37,2 | 39,9 | 41,2 | 43,4 | 42,2 | 42,6 | 40,6 | -7,9% |
| 10 | Ibagué | 3 | 38,5 | 37,2 | 35,4 | 35,1 | 36,3 | 37,2 | 36,7 | 36,4 | 37,8 | 37,2 | 37,8 | 39,1 | 1,6% |
| 11 | Bogotá | 3 | 36,5 | 37,1 | 36,2 | 35,2 | 34,9 | 35,5 | 36,3 | 37,4 | 37,3 | 37,4 | 40,7 | 40,2 | 10,1% |
| 12 | Pereira | 3 | 36,6 | 34,8 | 30,4 | 29,0 | 34,7 | 28,9 | 39,5 | 41,2 | 39,6 | 41,8 | 34,2 | 42,4 | 15,8% |
| 13 | Medellín | 3 | 37,6 | 36,6 | 38,3 | 37,4 | 36,2 | 38,3 | 37,7 | 40,4 | 36,7 | 38,1 | 37,4 | 38,3 | 1,9% |
| 14 | Bogotá | 3 | 36,9 | 34,2 | 33,6 | 33,3 | 31,5 | 32,0 | 29,7 | 29,3 | 26,4 | 26,9 | 26,8 | 25,9 | -29,8% |
| 15 | Medellín | 4 | 56,6 | 59,6 | 61,1 | 64,4 | 61,2 | 61,6 | 62,1 | 61,7 | 59,0 | 58,5 | 60,3 | 59,7 | 5,5% |
| 16 | Medellín | 4 | 55,8 | 56,1 | 55,4 | 51,1 | 53,8 | 53,8 | 56,0 | 54,4 | 55,4 | 56,3 | 54,5 | 52,5 | -5,9% |
| 17 | Bogotá | 4 | 49,9 | 49,0 | 47,6 | 50,6 | 48,1 | 50,4 | 48,0 | 47,7 | 49,5 | 48,1 | 48,7 | 49,8 | -0,2% |
| 18 | Pereira | 4 | 51,3 | 50,4 | 51,8 | 52,3 | 50,9 | 52,8 | 50,8 | 51,7 | 51,1 | 48,9 | 50,4 | 54,6 | 6,4% |
| 19 | Bogotá | 4 | 46,3 | 44,8 | 44,0 | 44,5 | 45,5 | 45,9 | 46,0 | 45,6 | 44,1 | 48,1 | 44,6 | 49,8 | 7,6% |
| 20 | Pereira | 4 | 50,5 | 48,4 | 49,6 | 49,6 | 47,7 | 50,7 | 48,7 | 48,4 | 47,6 | 47,2 | 48,1 | 47,6 | -5,7% |
| 21 | Bogotá | 4 | 52,5 | 49,5 | 49,5 | 48,9 | 49,6 | 49,2 | 47,8 | 46,5 | 45,7 | 46,9 | 48,3 | 46,9 | -10,7% |
| Mean | | | 48,7 | 47,8 | 47,5 | 47,2 | 47,3 | 47,3 | 46,8 | 47,1 | 47,1 | 47,4 | 47,7 | 48,2 | -1,0% |
| Total QT patients | | | 40319 | 39405 | 42568 | 41209 | 43524 | 41261 | 42673 | 43130 | 44378 | 44130 | 42169 | 42125 | 4,5% |
| Total patients attended | | | 84088 | 83436 | 90680 | 87478 | 93038 | 87980 | 92433 | 93236 | 95701 | 94754 | 88408 | 87825 | 4,4% |

*: Percentage of patients with at least one QT-prolonging drug. **: December vs January
